# NSAIDs Naproxen, Ibuprofen, Salicylate, and Aspirin Inhibit TRPM7 Channels by Cytosolic Acidification

**DOI:** 10.3389/fphys.2021.727549

**Published:** 2021-10-18

**Authors:** Rikki Chokshi, Orville Bennett, Tetyana Zhelay, J. Ashot Kozak

**Affiliations:** Department of Neuroscience, Cell Biology and Physiology, Boonshoft School of Medicine, College of Science and Mathematics, Wright State University, Dayton, OH, United States

**Keywords:** cation channel, acidification, pH, lymphocyte, phosphoinositide, analgesic, magnesium

## Abstract

Non-steroidal anti-inflammatory drugs (NSAIDs) are used for relieving pain and inflammation accompanying numerous disease states. The primary therapeutic mechanism of these widely used drugs is the inhibition of cyclooxygenase 1 and 2 (COX1, 2) enzymes that catalyze the conversion of arachidonic acid into prostaglandins. At higher doses, NSAIDs are used for prevention of certain types of cancer and as experimental treatments for Alzheimer’s disease. In the immune system, various NSAIDs have been reported to influence neutrophil function and lymphocyte proliferation, and affect ion channels and cellular calcium homeostasis. Transient receptor potential melastatin 7 (TRPM7) cation channels are highly expressed in T lymphocytes and are inhibited by Mg^2+^, acidic pH, and polyamines. Here, we report a novel effect of naproxen, ibuprofen, salicylate, and acetylsalicylate on TRPM7. At concentrations of 3–30mM, they reversibly inhibited TRPM7 channel currents. By measuring intracellular pH with the ratiometric indicator BCECF, we found that at 300μM to 30mM, these NSAIDs reversibly acidified the cytoplasm in a concentration-dependent manner, and propose that TRPM7 channel inhibition is a consequence of cytosolic acidification, rather than direct. NSAID inhibition of TRPM7 channels was slow, voltage-independent, and displayed use-dependence, increasing in potency upon repeated drug applications. The extent of channel inhibition by salicylate strongly depended on cellular PI(4,5)P_2_ levels, as revealed when this phospholipid was depleted with voltage-sensitive lipid phosphatase (VSP). Salicylate inhibited heterologously expressed wildtype TRPM7 channels but not the S1107R variant, which is insensitive to cytosolic pH, Mg^2+^, and PI(4,5)P_2_ depletion. NSAID-induced acidification was also observed in Schneider 2 cells from *Drosophila*, an organism that lacks orthologous COX genes, suggesting that this effect is unrelated to COX enzyme activity. A 24-h exposure to 300μM–10mM naproxen resulted in a concentration-dependent reduction in cell viability. In addition to TRPM7, the described NSAID effect would be expected to apply to other ion channels and transporters sensitive to intracellular pH.

## Introduction

Non-steroidal anti-inflammatory drugs (NSAIDs) are widely used as medication for mild and moderate pain in diseases such as rheumatoid arthritis, spondyloarthropathies and gout (Rider and Jordan, 2010; Firth, 2012). The mechanism of their analgesic action is the inhibition of cyclooxygenase enzymes COX1 and COX2, which catalyze the production of prostaglandins responsible for the pain and inflammation symptoms (Vane, 1971; Fitzpatrick, 2004; Burke et al., 2006). NSAIDs, therefore, exert their therapeutic effects by markedly reducing the production of prostaglandins. The original NSAIDs were nonselective inhibitors of COX enzymes and were followed by compounds specific for COX2, the COX enzyme inducible by inflammation in macrophages, monocytes, and other cell types (Burke et al., 2006). Adverse effects of nonselective NSAIDs include peptic ulcer disease, gastrointestinal bleeding, aseptic meningitis, hyperkalemia, urticaria/angioedema, and aspirin-exacerbated respiratory disease (AERD), and are thought to be mediated by the constitutively active COX-1 enzyme (Berges-Gimeno et al., 2002; Woessner, 2003; Kong et al., 2007). High doses of aspirin and salicylate (millimolar range) have long been known to cause tinnitus, hearing loss and cochlea degeneration (Wei et al., 2010; Namikawa et al., 2017).

Over the years, it has become clear that COX enzymes are not the only target of NSAIDs (Tegeder et al., 2001; Bishay et al., 2010). Moreover, in addition to their widespread use as pain relievers, NSAIDs treat numerous other conditions. Several lines of evidence suggest that NSAIDs can be useful in Alzheimer’s disease (AD) by reducing inflammation in the brain (Lim et al., 2000; McGeer and McGeer, 2001b; Aisen et al., 2003; Trepanier and Milgram, 2010), and were found to decrease the incidence of AD in more than a dozen epidemiological studies (McGeer and McGeer, 2001a). NSAIDs have also been reported to reduce colon cancer in animal models of this disease, and the mechanisms may involve reduced proliferation and increased apoptosis of cancer cells (Shiff et al., 1995, 1996; Shiff and Rigas, 1999; Villalobos et al., 2017). Importantly, the beneficial effects of NSAIDs in these diseases were found at doses substantially higher than necessary to inhibit COX enzymes (Tegeder et al., 2001; Schonthal, 2010).

NSAIDs inhibit several ion channels independently of their COX-targeting effects. ASIC (acid-sensing ion channel), Nav1 (voltage-gated sodium channel), TRPV1, and TRPA1, channels involved in peripheral nociception, are inhibited by NSAIDs, which possibly contributes to their analgesic action (Voilley, 2004; Park et al., 2007; Wang et al., 2012; Nozadze et al., 2016; Tsagareli et al., 2018). ATP-sensitive potassium channels (Kir6.2) in pancreatic β cells are inhibited by meclofenamic acid resulting in elevated intracellular calcium and insulin secretion (Li et al., 2007). *Drosophila* and rat voltage-gated potassium channels (Kv2) were directly inhibited by celecoxib (active ingredient of Celebrex) at micromolar concentrations, causing low beating rate and arrhythmia in cultured cardiomyocytes and reducing spontaneous firing in retinal neurons (Frolov et al., 2008a,b).

TRPM7 is a dual ion channel/protein kinase, highly expressed in cells of the immune system such as T and B lymphocytes, basophils, mast cells, macrophages and microglia (Kozak et al., 2002; Jiang et al., 2003; Chokshi et al., 2012b; Freichel et al., 2012; Huang et al., 2014; Kaitsuka et al., 2014; Krishnamoorthy et al., 2018). TRPM7 channels conduct cations and are unique in that they require depletion of cytosolic Mg^2+^ for opening (Hermosura et al., 2002; Kozak et al., 2002; Prakriya and Lewis, 2002). In whole-cell patch clamp, it takes several minutes of internal perfusion of metal chelators to achieve full activation of TRPM7 currents. Conversely, inclusion of 300μM and higher concentrations of Mg^2+^ in internal solutions will prevent current development (Chokshi et al., 2012a). We recently discovered that in intact cells TRPM7 channel activation and inhibition can be achieved by prolonged exposure of cells to low (micromolar) and high (millimolar) external [Mg^2+^], respectively (Mellott et al., 2020). In addition to Mg^2+^, other metal ions, polyamines and protons, are also inhibitory (Kozak and Cahalan, 2003; Kozak et al., 2005; Chokshi et al., 2012b; Zhelay et al., 2018). Channel inhibition by Mg^2+^ and protons does not depend on the kinase activity (Kozak et al., 2005; Matsushita et al., 2005; Beesetty et al., 2021). TRPM7 channels have been implicated in cellular and body Mg^2+^ homeostasis (Zou et al., 2019). TRPM7 channel activity is required for Mg^2+^ entry in T cells; however, the mechanism of Mg^2+^ permeation is not fully elucidated (Deason-Towne et al., 2011; Chubanov et al., 2018; Mellott et al., 2020).

Naproxen, ibuprofen, and salicylates belong to the aryl propionic acid derivative group. We previously reported that TRPM7 channels are inhibited reversibly by propionate (Kozak et al., 2005). The mechanism of this effect is cytosolic acidification; accordingly, in the presence of divalent cations, TRPM7 channels are inhibited by acidic pH with pH_50_ of 6.3 (Chokshi et al., 2012b). The inhibition reflects interference with channel stimulation by the phospholipid PI(4,5)P_2_ (PIP_2_) present in the plasma membrane (Kozak et al., 2005; Zhelay et al., 2018). Moreover, serine 1,107 substitutions with bulkier residues in TRPM7 greatly diminished inhibition by protons, Mg^2+^, and polyamines while increasing its sensitivity to the agonist PIP_2_ (Zhelay et al., 2018).

Here, we have characterized the effects of NSAIDs acetylsalicylate, salicylate, naproxen, and ibuprofen on TRPM7 channels. We found that these drugs potently inhibit the native TRPM7 currents in Jurkat T cells. At 3–30mM the inhibition was reversible with a slow onset and offset, requiring several minutes. Repeated applications of the drugs in the same cell resulted in progressively stronger reduction in the current, showing use-dependence or sensitization. Single-cell pH measurements showed that the drugs acidified the Jurkat cytosol, and this effect was concentration-dependent. Long-term exposure to naproxen reduced cell viability to a greater extent than salicylate. Depletion of PIP_2_ in HEK293 cells by expressing *Ciona* voltage-sensitive lipid phosphatase (CiVSP) resulted in significantly stronger channel inhibition by salicylate. Moreover, salicylate inhibited heterologously expressed wildtype TRPM7 channels but not the pH and Mg^2+^-insensitive variant S1107R. Based on these findings, we propose that the mechanism of TRPM7 channel inhibition by NSAIDs is acidification and interference with channel-PIP_2_ interactions. In *Drosophila* S2 cells which lack COX enzyme orthologs, we found a similar acidification induced by NSAIDs, demonstrating that COX enzymes are not involved in this process.

## Materials and Methods

### Cell Culture and Transfection

Human leukemic Jurkat T lymphocytes and human embryonic kidney (HEK293) cells were kept in a CO_2_ incubator (Thermo Scientific, Fairlawn, NJ, United States and Forma Scientific, Marietta, OH, United States) at 37°C and grown in RPMI-1640 (Lonza, Wakersville, MD, United States) supplemented with glutamine, 10% fetal bovine serum (FBS; BioWest, Riverside, MO, United States), and penicillin/streptomycin (Thermo; Chokshi et al., 2012b; Zhelay et al., 2018). Jurkat T cells were passaged by diluting the cell suspension in the complete culture medium, and HEK293 cells were passaged using Cellstripper non-enzymatic cell dissociation solution (Mediatech, Manassas, VA, United States). HEK cells were transfected with pEGFP-TRPM7, pEGFP-TRPM7 S1107R, pIRES2-EGFP CiVSP, and pIRES2-EGFP CiVSP C363S plasmids using TransIT-LT1 transfection reagent (Mirus Bio, Madison, WI, United States) as previously described in detail (Zhelay et al., 2018). Electrophysiological recordings were made 1–2days after transfection. Successfully transfected cells were identified by their GFP fluorescence. *Drosophila* Schneider 2 (S2) cells from Drosophila Genomics Resource Center (Bloomington, IN, United States) were maintained at room temperature and grown in Schneider’s insect medium (Lonza) supplemented with 10% fetal bovine serum (Cherbas and Cherbas, 2007). For passaging, cells were mechanically lifted and diluted in fresh culture medium before re-plating.

### Patch-Clamp Electrophysiology

Patch-clamp recordings in the whole-cell configuration were performed as previously described (Kozak and Cahalan, 2003; Chokshi et al., 2012b; Zhelay et al., 2018). Briefly, monovalent TRPM7 currents were evoked using a Mg^2+^-free pipette solution which contained (in mM): 112 Cs or K glutamate, 8 NaCl, 0.09 CaCl_2_, 12 EGTA, 1 HEPES, and pH 7.3. In some experiments, 250μM MgCl_2_ was added to this solution, yielding free [Mg^2+^] of 134μM as estimated by Webmaxc.^1^ Extracellular (bath) solution was composed of (in mM) 140 Cs or Na aspartate, 3 CsCl, 4 HEDTA, 10 HEPES, and pH 7.3. In divalent metal-free (DVF) external solutions, TRPM7 current–voltage (I–V) relation becomes semi-linear (Kozak et al., 2002), reversing close to 0mV. Recordings were made using the computer-driven EPC-10 patch-clamp amplifier (HEKA Elektronik, Lambrecht, Germany) and PatchMaster (v. 2.6) software. Instantaneous I–V relations were obtained from command voltage ramps of 211ms duration spanning −100mV or−85mV to +85mV applied every 2.5 or 1.5s (Chokshi et al., 2012b). Currents were sampled at 5kHz and low-pass filtered at 2.9kHz. Current amplitudes usually reached a maximum 3–5min after break-in (Chokshi et al., 2012b). Naproxen sodium, ibuprofen sodium, salicylic acid, and acetylsalicylic acid (Sigma-Aldrich, St. Louis, MO, United States and Acros Organics, Geel, Belgium) were dissolved freshly in the extracellular solution and the pH measured on the day of experiment. Osmolalities of the drug-containing solutions were adjusted by reducing cesium/sodium aspartate concentrations accordingly and measured with a freezing point depression osmometer (Precision Systems, Natick, MA, United States). Both the control and drug-containing external solutions had equal concentrations of permeant monovalent cations (Cs^+^ and Na^+^). The patch pipettes were manufactured on a DMZ-Universal (Zeitz Instruments, Martinsried, Germany) and P-1000 (Sutter Instrument, Novato, CA, United States) horizontal pullers from borosilicate glass capillaries (Warner Instruments, Hamden, CT, United States) and fire-polished on a MF-830 microforge (Narishige, Tokyo, Japan). Drug-containing solutions were applied to the recording chamber using gravity-fed rapid perfusion systems SB-77 (Warner Instruments, Hamden, CT, United States) and ValveLink 8.2 (AutoMate Scientific, Berkeley, CA, United States), respectively, or slow bath perfusion. All drugs were tested with slow and rapid perfusion, yielding indistinguishable results. Data were analyzed and plotted using Origin (v. 8 and 2016) software (OriginLab, Northampton, MA, United States). Patch clamp experiments were performed at room temperature. Student’s two-sample t test was used for determining statistical significance.

### Single-Cell pH Measurement

For intracellular pH imaging experiments, 35-mm glass-bottom imaging chambers were used with solution volume of ~1ml. Cells were seeded on these chambers and incubated in 2μM BCECF-AM (Invitrogen, Carlsbad, CA, United States) containing buffer for ~45min at room temperature (Chokshi et al., 2012a). The fluorescent dye-containing solution was aspirated and replaced with normal external solution composed of (in mM): 2 CaCl_2_, 4.5 KCl, 140 NaCl, 10 HEPES-Na^+^, 10 glucose, and pH 7.3. The imaging chamber with attached cells was then mounted on the movable stage of an inverted microscope (Olympus, Tokyo, Japan). Ratiometric imaging was performed by illuminating cells in a selected field every 12s at 490 and 440nm using a Lambda 10B shutter and filter wheel (Sutter). Fluorescence was measured at 510nm. The light source was a 175W Xenon lamp (QED, Lexington, KY, United States). Images were taken and processed with Pixelfly CCD camera (PCO. Imaging, Kelheim, Germany) and InCytIM 2 software (Intracellular Imaging, Cincinnati, OH, United States). Emitted light intensities averaged for individual cells in the imaging field were plotted against time using Origin. Solutions in the imaging chamber were exchanged with a syringe-driven plastic tubing perfusion system. Typically, at least 20ml of each solution was perfused through the ~1ml volume chamber to ensure a complete solution exchange. The high [K^+^] solution contained 130mM KCl, 20mM NaCl, 1mM CaCl_2_, 0.5mM MgSO_4_, 1mM NaH_2_PO_4_, 10mM HEPES, and 5mM glucose. Nigericin (Calbiochem, La Jolla, CA, United States) was added to this solution at 2–10μM. The ratio value was taken at time points where pH_i_ had achieved a steady value. Experiments were performed at room temperature.

### Cytotoxicity Assay

For estimating drug cytotoxicity, Jurkat T cells were maintained in T25 cell culture flasks in RPMI-1640 containing glutamine and 25mM HEPES (Hyclone, Logan, UT, United States) supplemented with 10% FBS and penicillin/streptomycin. NSAID powder was added on the day of experiment to yield the indicated final concentrations (Supplementary Figure S1), and cells were collected after 24-h incubation in a CO_2_ incubator at 37°C. Cell viability was measured by trypan blue exclusion using a Vi-CELL automated cell counter (Beckman Coulter, Brea, CA, United States) as previously described in detail (Gibson et al., 2016; Mellott et al., 2020). For each concentration of the drug, the experiment was repeated with three different groups of cells.

## Results

### NSAIDs Naproxen, Salicylic Acid, Acetylsalicylic Acid, and Ibuprofen Reversibly Inhibit Native TRPM7 Channels in Jurkat T Lymphocytes

We tested naproxen, ibuprofen, salicylic acid, and acetylsalicylic acid for their ability to inhibit the endogenous TRPM7 channel current in Jurkat T lymphocytes. As described in Materials and Methods, we allowed several minutes of Mg^2+^ depletion for the TRPM7 current to develop to full extent before applying the drugs.

**Figure 1:**
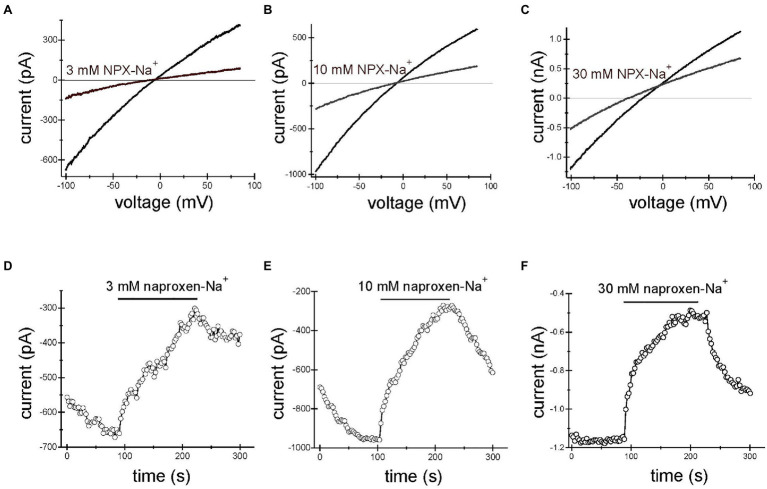
Naproxen-Na^+^ inhibits TRPM7 current. TRPM7 monovalent I–V relations in the absence and presence of 3mM **(A)**, 10mM **(B)** and 30mM **(C)** naproxen. **(D–F)** Depict the time courses of inhibition by 3mM **(D)**, 10mM **(E)**, and 30mM **(F)** naproxen. The presence of drug is indicated by horizontal bars. Inward current amplitude was measured at −100mV and plotted against time. Representative whole cell patch-clamp recordings from *n*=10 **(A,D)**, *n*=8 **(B,E)**, and *n*=4 **(C,F)** Jurkat T cells. Here and in Figures 2, 3, the drug was applied multiple times. The initial current development after break-in is not shown. The internal and external solutions were K^+^ and Na^+^ based, respectively.

Application of naproxen-Na^+^ at concentrations of 3, 10, and 30mM reproducibly inhibited TRPM7 currents. Figures 1A–C show the effect of 3, 10, and 30mM naproxen on the I–V relation of the monovalent TRPM7 current in three representative Jurkat T lymphocytes. The current magnitude was reduced by 3mM (A), 10mM (B), and 30mM (C) naproxen in a voltage-independent manner. In Figures 1D–F, the time courses of inhibition by the indicated concentration of naproxen are shown. We used low concentrations of pH buffer HEPES in these recordings (see below). The onset of inhibition was slow, reaching a steady state in approximately 2min. Washout of drug effect was equally slow (Figures 1D–F). In most cells, this inhibition was reversible (Figures 1D–F), but in some cells at higher concentrations, inhibition could not be reversed even after prolonged washing, presumably because the TRPM7 channels had run down (Kozak et al., 2002; Chokshi et al., 2012b).

**Figure 2:**
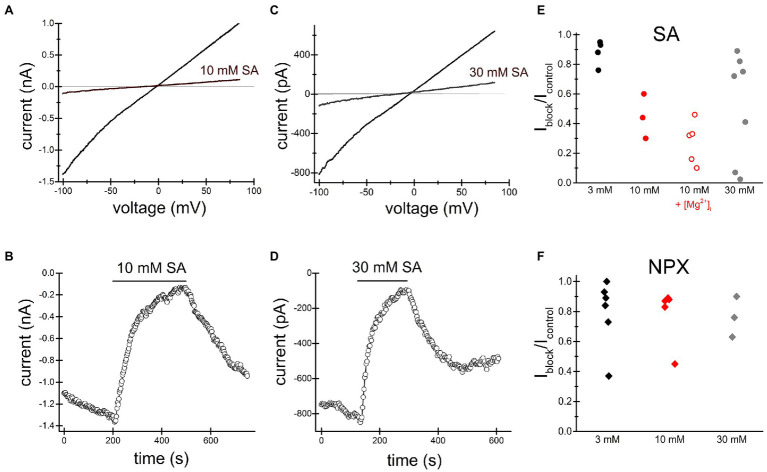
Salicylate inhibits TRPM7 current. Summary of naproxen and salicylate inhibition of TRPM7 current at various concentrations. TRPM7 monovalent I–V relations obtained in the absence and presence of 10mM **(A)** and 30mM **(C)** salicylate. **(B,D)** show the corresponding time course of salicylate inhibition. Representative recordings from *n*=3 **(A,B)** and *n*=9 **(C,D)** Jurkat T cells. Third **(A,B)** and fourth **(C,D)** drug applications are depicted. **(E)** Summary of salicylate effects on TRPM7 current at 3, 10, and 30mM. Each symbol represents the ratio of current amplitude after inhibition (I_block_) to current amplitude before drug application (I_control_) in one cell. The internal solution contained 1mM HEPES. In cells represented by empty red circles, the internal solution was supplemented with 250μM MgCl_2_ (free [Mg^2+^]=134μM). **(F)** Summary of naproxen effects on TRPM7 current at 3, 10, and 30mM. The internal solution contained 1mM HEPES and no added Mg^2+^. In **(E,F)** responses to the second application of the drug are plotted.

**Figure 3:**
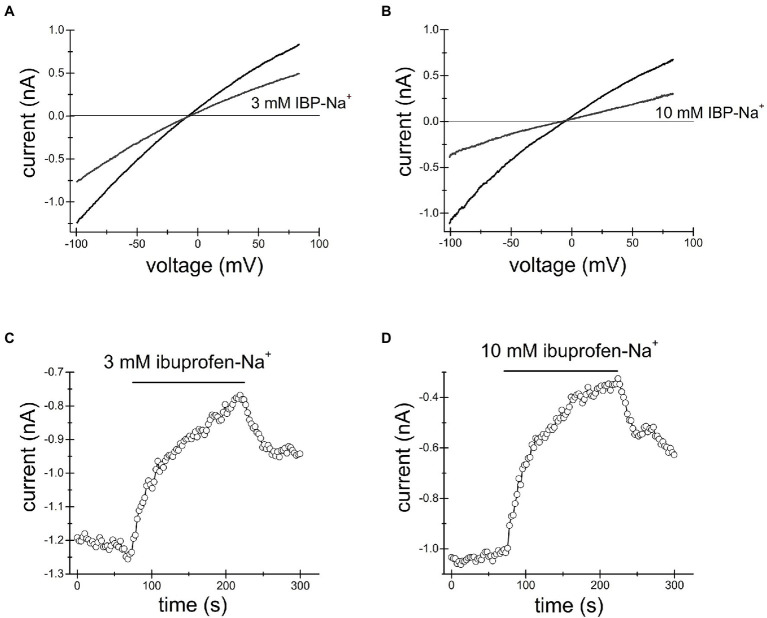
Ibuprofen-Na^+^ inhibits TRPM7 current. TRPM7 monovalent I–V relations obtained in the absence and presence of 3mM **(A)** and 10mM **(B)** ibuprofen. **(C,D)** show the time course of ibuprofen effect in the same cells. Current amplitudes were measured at −100mV and plotted against time. Representative recordings from *n*=7 **(A,C)** and *n*=3 **(B,D)** Jurkat T cells.

Figures 2, 3 show the effects of 3, 10mM ibuprofen-Na^+^ and salicylate in Jurkat cells. Similar to naproxen, these drugs reversibly inhibited native TRPM7 currents. The extent of current inhibition varied greatly from cell to cell showing no apparent dependence on concentration of the drug. Adding Mg^2+^ to the internal solution appeared to facilitate current inhibition (Figure 3E). Moreover, repeated application of the same concentration of drug resulted in more robust inhibition (see below).

### NSAIDs Naproxen, Ibuprofen, and Salicylate Acidify the Cytoplasm

Since intracellular acidic pH potently and reversibly inhibits TRPM7 channels (Kozak et al., 2005; Chokshi et al., 2012b; Zhelay et al., 2018), and the NSAIDs in question are weak acids, we hypothesized that at relevant concentrations (Jung and Schwartz, 1994; Huntjens et al., 2006; Capone et al., 2007b), NSAIDs acidify the cell cytoplasm and inhibit the channels indirectly, by exposing them to lower pH from inside. We previously found that 2-APB inhibits TRPM7 channels by acidifying the cytoplasm (Chokshi et al., 2012a).

#### Naproxen

**Figure 4:**
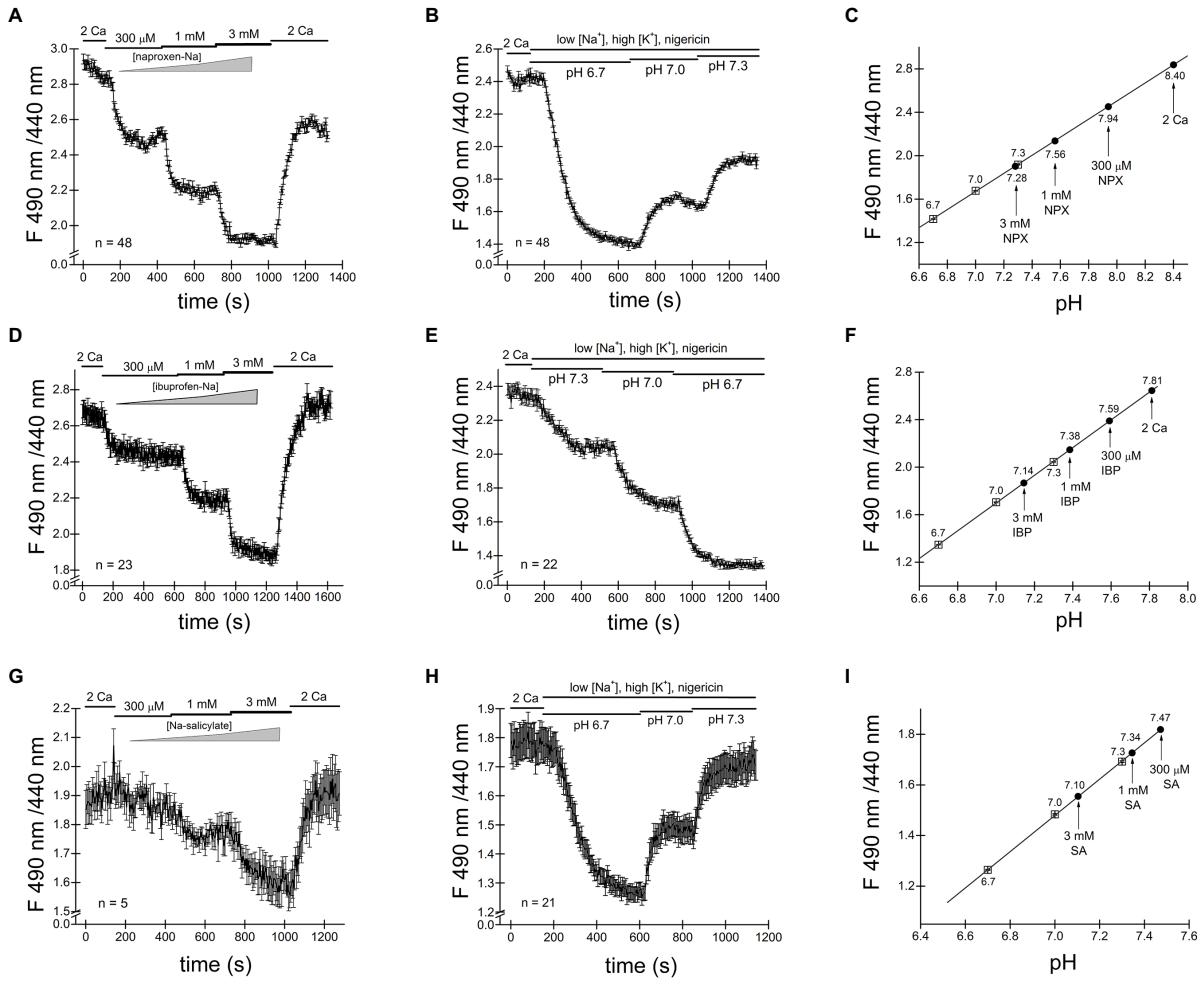
Cytosolic acidification induced by naproxen, ibuprofen and salicylate. Fluorescence ratiometric pH measurements were performed in Jurkat T cells loaded with BCECF dye and superfused with the indicated concentrations of naproxen **(A)**. Naproxen-induced acidification was reversed upon washout (2 Ca bar). **(B)** Calibration experiment was performed by exposing the cells to low Na^+^/high K^+^ solution containing 5μM nigericin at three different extracellular pH values (6.7, 7.0, and 7.3). **(C)** Calibration curve constructed from measurement means taken at steady state in **(B)**. The line is a linear fit. Mean steady state fluorescence ratios in the presence of naproxen from (**A**; indicated by vertical arrows) are superimposed on the calibration curve to determine the corresponding cellular pH. **(D,G)** Ratiometric BCECF measurements for ibuprofen and salicylate were performed as in **(A–C)**. Ibuprofen and salicylate dose-dependently and reversibly acidified the Jurkat T cell cytoplasm. Data points in **(A,B,D,E,G,H)** represent means±SEM obtained from measurements performed in groups of cells. **(F,I)** Calibration curves generated from **(D,E)**, and **(G,H)**, respectively, as in **(C)**.

We loaded Jurkat T cells with the fluorescent dye BCECF for pH measurement as described in detail in Materials and Methods and (O’Connor and Silver, 2007; Chokshi et al., 2012a). Figure 4A shows single-cell pH_i_ time course during sequential exposure to 300μM, 1mM and 3mM naproxen. Increasing drug concentration resulted in progressively more acidic cytoplasmic pH. In order to estimate the actual intracellular pH values achieved in the presence of each naproxen concentration, we performed calibration experiments by bathing the cells in high K^+^ (130mM) solutions of known pH containing H^+^/K^+^ antiporter nigericin (Negulescu and Machen, 1990; Bowser et al., 1999). In Figure 4B, the cells were first incubated in normal (low K^+^, high Na^+^) solution at pH 7.3 to collect baseline ratiometric measurements. The bathing solution was then switched to high K^+^ solutions at pH 6.7, 7.0, and 7.3, with nigericin, and corresponding ratio changes were recorded. pH 6.7 in the presence of nigericin resulted in a slow drop in ratiometric signal reaching a plateau in approximately 5min. As expected, pH 7.0 and pH 7.3 solutions brought the ratiometric signals to higher values. In Figure 4C, the calibration curve generated from measurements in Figure 4B is shown: average ratio values at steady state (squares) were plotted against extracellular pH and could be fitted with a linear equation. We superimposed the ratio values obtained at steady state in Figure 4A for naproxen concentrations of 300μM, 1mM, and 3mM on the calibration line (circles). We obtained cytoplasmic pH values of 7.94, 7.56, and 7.28, respectively, for these concentrations of the drug. Thus, we find that naproxen, at therapeutically relevant concentrations, acidifies Jurkat T cell cytoplasm in a concentration-dependent manner.

#### Ibuprofen

We proceeded to measure pH changes induced by commonly used NSAID drug ibuprofen in Jurkat T cells. In the experiment shown in Figure 4D BCECF loaded cells were incubated in normal external solution with addition of increasing concentrations of ibuprofen. Figure 4D shows that ibuprofen reduced the ratiometric signal in a dose-dependent manner at 300μM, 1mM, and 3mM. Reductions in ratiometric signal were reversed upon washout (2 Ca bar). We performed calibration experiments similar to Figure 4B and superimposed the steady-state ratio measurements from Figure 4D on the calibration line obtaining values of 7.59, 7.38, and 7.14 for 300μM, 1mM, and 3mM ibuprofen, respectively. We find that like naproxen, ibuprofen significantly acidifies Jurkat T cell cytoplasm concentration-dependently and that the effect is reversible.

#### Salicylate

In experiments similar to Figures 4A–F, we tested the effect of salicylate on cytosolic pH. Addition of salicylic acid at 300μM, 1mM, and 3mM concentrations reduced the cell pH to 7.47, 7.34, and 7.10, respectively (Figures 4G–I). Similar to naproxen and ibuprofen, salicylate acidified Jurkat T cells in a concentration-dependent manner. Interestingly, there was no significant recovery from acidification in the presence of these drugs (Figure 4).

In the course of these experiments, we noticed that the resting pH values between various groups of Jurkat T cells varied greatly. In Figure 4C, for example, the initial pH appears to be alkaline, close to 8.4. In cells shown in Figure 4F, the resting pH is close to 7.81 and in Figure 4I, it is 7.47. The nature of this variability in cytosolic pH between various batches of cells is not known and necessitated calibration in the same group of cells exposed to drugs. Based on our intracellular pH measurements, we conclude that the substantial cytosolic acidification caused by NSAIDs tested can explain inhibition of TRPM7 channels observed in patch-clamp electrophysiology. The cell-to-cell variability of resting pH_i_ may explain the variability in channel inhibition (see above).

### Does the NSAID Effect Involve the COX Pathway?

**Figure 5:**
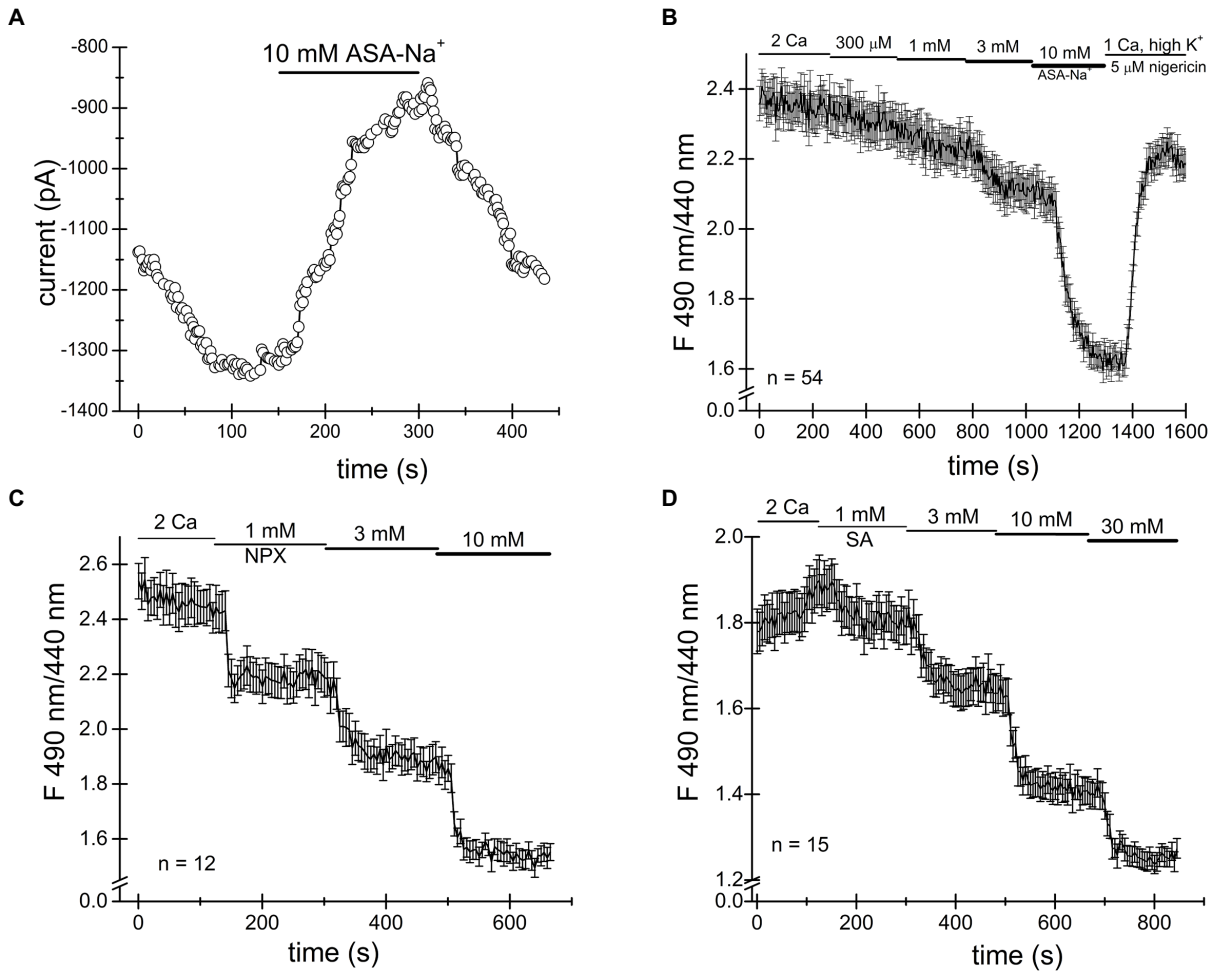
Cytosolic acidification caused by NSAIDs does not require COX enzymes. **(A)** Acetylsalicylate inhibits TRPM7 current in Jurkat T cells reversibly. **(B)** Acidification in Jurkat T cells induced by acetylsalicylate is reversible. Naproxen **(C)** and ibuprofen **(D)** acidify *Drosophila* S2 cells.

Ibuprofen, naproxen and salicylic acid are thought to involve a reversible inhibition of COX enzymes by binding non-covalently (Burke et al., 2006). As shown in Figures 1–4, both TRPM7 channel inhibition and cytosolic acidification are reversible upon removal of the drug, and therefore may involve COX enzyme activity. Acetylsalicylic acid (aspirin), on the other hand, inhibits these enzymes irreversibly, by acetylation. We, therefore, tested acetylsalicylate for its ability to acidify the cytosol. Figures 5A,B shows that similar to naproxen ibuprofen and salicylate, acetylsalicylate-Na^+^ reversibly inhibited TRPM7 channels and acidified the cytosolic pH in a concentration-dependent manner. Acidification was reversed upon washout. Acetylsalicylate acetylates a serine in COX-1 and 2 in the active site of the enzyme. Since it stands out among other NSAIDs for the irreversibility of its COX inhibition, reversibility of channel acidification and channel inhibition strongly suggests that NSAID effect on cytosolic pH is independent of their effect on COX enzymes.

It has been reported that *Drosophila* genome lacks orthologs of mammalian COX enzymes and TRPM7 channel/kinase (Manning et al., 2002; Frolov et al., 2008b). In order to further test whether the NSAID acidifying effect on cytosol requires COX enzymes, we performed intracellular pH measurements in *Drosophila* S2 cells. As shown in Figures 5C,D, we find that naproxen and salicylate elicited reversible acidifications of the cytoplasm, qualitatively similar to their effect in Jurkat T cells. This experiment confirmed that neither COX enzymes nor, apparently, TRPM7 are required for the acidification caused by these NSAIDs.

### Salicylate and Naproxen Inhibition of TRPM7 Current Depends on PIP_2_ Levels

**Figure 6:**
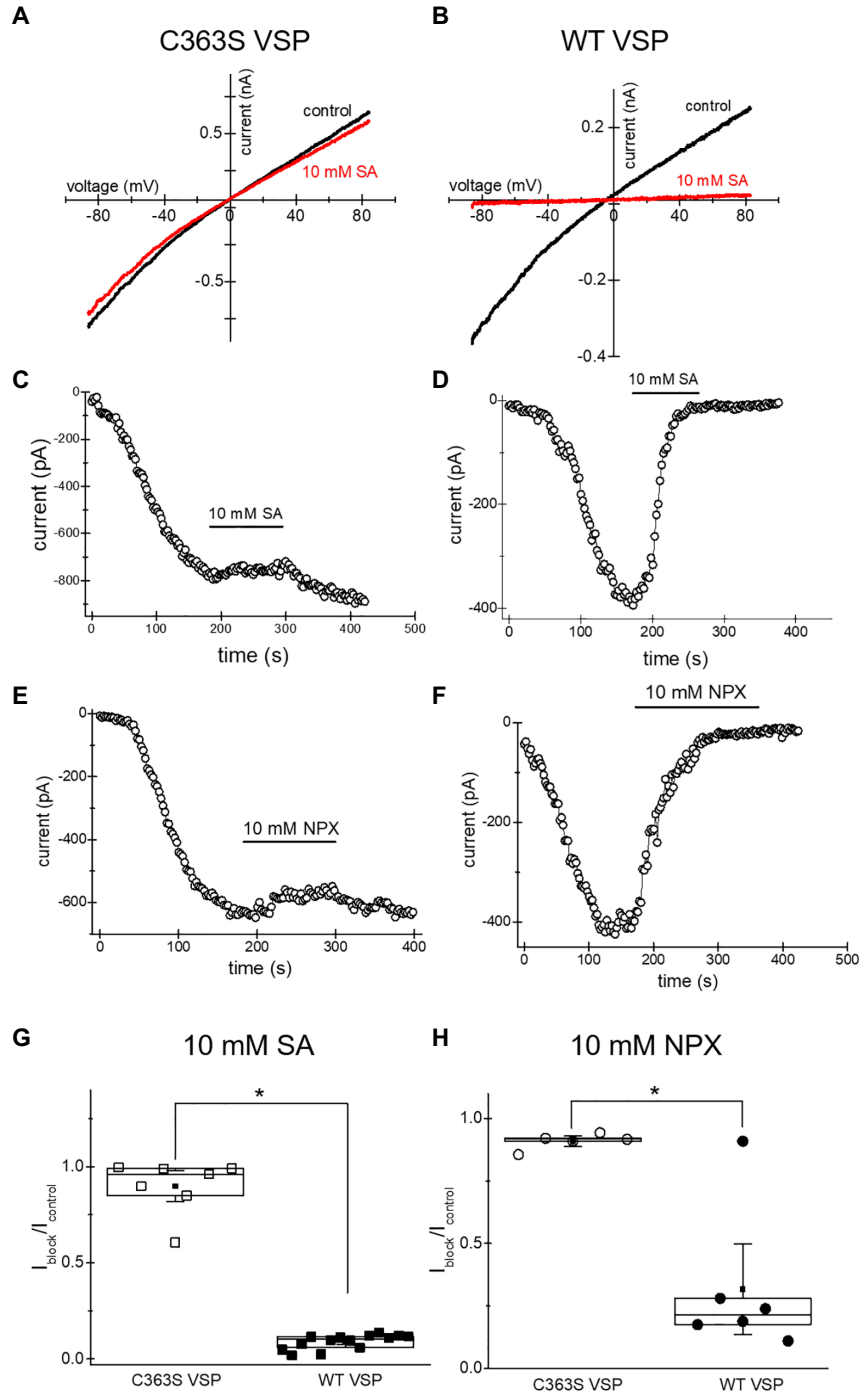
Salicylate effect on TRPM7 current in HEK293 cells expressing WT CiVSP and its inactive C363S variant. **(A,B)** Endogenous TRPM7 monovalent I–V relations in the absence and presence of 10mM salicylate. HEK293 cells were transfected with C363S VSP **(A)** and WT VSP **(B)**. **(C,D)** Time courses of 10mM salicylate effect in HEK cells expressing C363S and WT VSP. **(E,F)** Time courses of 10mM naproxen effect in HEK cells overexpressing C363S and WT VSP. **(G,H)** I_block_/I_control_ ratios measured for 10mM salicylate **(G)** and 10mM naproxen **(H)**. Current amplitude was measured at the 40th **(G)** and 35th **(H)** ramp during drug application (I_block_) and divided by the amplitude immediately before drug application (I_control_). The current amplitudes were measured at −85mV. The internal and external recording solutions were Cs^+^ based. ^*^p <0.05, Student’s two-sample t test.

The inhibition of TRPM7 channels by cytosolic Mg^2+^, polyamines, and protons is indirect and involves the electrostatic screening/sequestration of phosphoinositide PIP_2_ (Kozak et al., 2005; Chokshi et al., 2012c; Zhelay et al., 2018). PIP_2_ is a necessary cofactor of TRPM7 and other TRP channels (reviewed in Suh and Hille, 2008; Rohacs, 2014). The voltage-sensitive lipid phosphatase (VSP) from Ciona is a convenient tool for depleting phosphoinositides in intact cells (Yudin et al., 2021). We showed previously that depletion of PIP_2_ by overexpressing VSP mimicked inhibition of TRPM7 by cytosolic cations (Zhelay et al., 2018). Specifically, inhibition by acidic pH and 2-APB, a TRPM7 blocker which acidifies the cytosol, was strengthened in PIP_2_-depleted cells (Chokshi et al., 2012a; Zhelay et al., 2018). In order to examine if TRPM7 channel inhibition by NSAIDs occurs by a similar mechanism, we measured salicylate and naproxen effects on the native TRPM7 current in HEK cells transfected with WT and C363S VSP. HEK cells possess substantial TRPM7 channel activity (Chokshi et al., 2012b). Ten millimolar salicylate and naproxen effect in cells expressing the inactive VSP mutant was very small (Figure 6). By contrast, both drugs inhibited TRPM7 currents completely in PIP_2_ depleted cells (Figure 6). It should be noted that in WT VSP expressing cells, drug washout could not be observed (Figures 6D,F), likely because of increased current rundown (Zhelay et al., 2018). These experiments strongly suggested that NSAID-induced TRPM7 current inhibition depends on cellular PIP_2_ levels, as found previously for 2-APB.

### Use-Dependence (Sensitization) of Channel Inhibition

**Figure 7:**
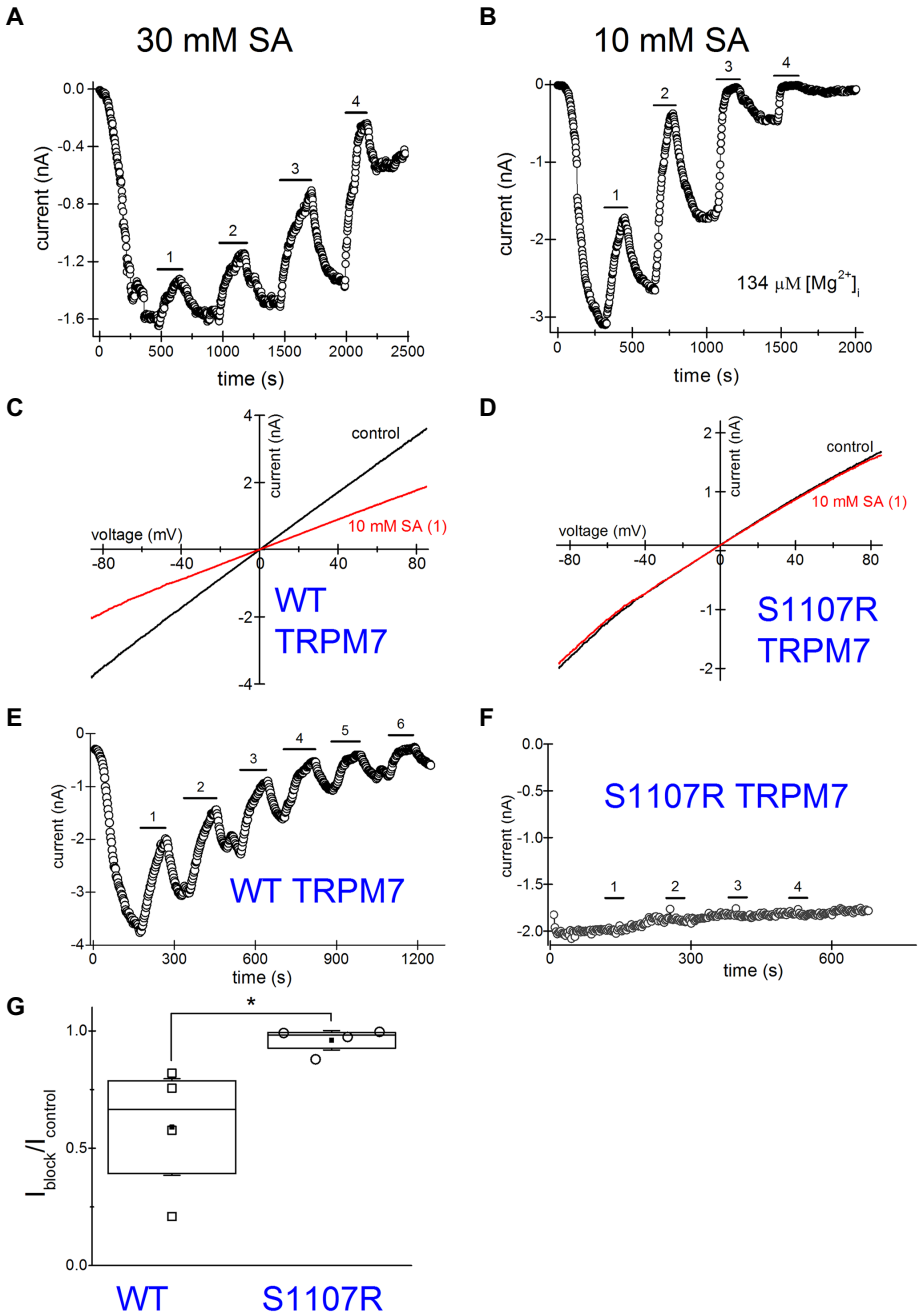
Increased inhibition of TRPM7 current revealed by multiple applications of salicylate. Salicylate effect on WT and S1107R TRPM7. **(A)** Effect of repeated application of 30mM salicylate on the native TRPM7 current in Jurkat T cells. The internal solution contained no added Mg^2+^. **(B)** Repeated application of 10mM salicylate in a Jurkat T cell. The internal solution contained 134μM free [Mg^2+^]. **(C,D)** I–V relations obtained in the absence and presence of 10mM salicylate (first application) in HEK293 cells expressing WT **(C)** and S1107R **(D)** mTRPM7. **(E,F)** The time course of repeated salicylate applications in the same experiments as **(C,D)**, respectively. **(G)** Fraction of current block by 10mM salicylate of WT and S1107R mutant mTRPM7 channels during second application of the drug (see **E,F**). Current amplitude was measured at the 30^th^ ramp during drug application (I_block_) and divided by the amplitude immediately before drug application (I_control_). The current amplitudes were measured at −85mV. The internal and external recording solutions were Cs^+^ based. ^*^p <0.05, Student’s pairwise t test.

Next, we investigated NSAID effects when applied repeatedly. Such drug application protocols with washout steps between them could mimic the situation *in vivo*, where the patient takes the drug several times daily and the effective concentrations of NSAID in the blood vary depending on proximity to the time of drug intake. Figure 7A shows a representative graph where 30mM salicylate was repeatedly applied. Inhibition was progressively more pronounced for every application. At the same time, the current was running down (amplitude of current after drug washout is smaller than before its application; see Kozak et al., 2005 for a detailed discussion of rundown).

TRPM7 is strongly dependent on intracellular free Mg^2+^ being inhibited by this ion with a double dose–response relationship with IC_50_ values of 10μM and 165μM (Chokshi et al., 2012b). Despite this, the majority of Jurkat T cells exhibit some degree of TRPM7 channel preactivation even without prior Mg^2+^ depletion (Chokshi et al., 2012b; Zhelay et al., 2018). This was observed in other cell types as well (Kozak and Cahalan, 2003). We performed a series of experiments with pipette solutions containing 134μM free [Mg^2+^]. Our goal was to compare NSAID inhibition in the presence and absence of intracellular Mg^2+^, since normally in intact T cells free Mg^2+^ concentration is close to 1mM (Rink et al., 1982; Ng et al., 1991). Figure 7B shows repeated application of 10mM salicylate with intracellular Mg^2+^ present. The main difference from Figure 7A was the increased speed of inhibition and rundown with Mg^2+^ inside. The current completely ran down in ~30min. Because of the fast rundown, it was impossible to completely reverse the inhibition. Also, it was difficult to assess the extent of block at third or fourth applications as it was already maximal but clearly, the second application of the drug was almost twofold more potent than the first one. These experiments suggested that use-dependence of NSAID effect is increased with Mg^2+^ inside, which was reminiscent of inhibition by propionate (Zhelay et al., 2018). In conclusion, both the extent of inhibition by NSAIDs and the speed of action demonstrate strong use-dependence.

We reasoned that increased inhibition during repeated application of NSAIDs could potentially be due to increased acidification caused by repeated application of these drugs. To test this possibility, we set out to compare the pH_i_ during repeated application of the drugs. As shown in Supplementary Figure S1A, ibuprofen was applied repeatedly to Jurkat T cells loaded with BCECF dye and corresponding pH-dependent fluorescence signal measured. The degree of acidification caused by this drug appeared similar for each application. This experiment demonstrates that the use-dependence of TRPM7 inhibition discussed in Figures 7A,B cannot be explained by greater acidification with repeated drug applications but must have a different underlying mechanism. This mechanism might be PIP_2_-dependent, as we found for propionate and 2-APB (Zhelay et al., 2018).

### Salicylate Effect on WT and S1107R Mutant TRM7 Channels

Several Ser1107 substitutions in TRPM7 result in significantly reduced sensitivity to Mg^2+^, acidic pH, and polyamines as well as PIP_2_ depletion (Hofmann et al., 2014; Zhelay et al., 2018). If TRPM7 channel inhibition by NSAIDs is a consequence of cytosolic acidification, then TRPM7 S1107R mutant, which is less pH-sensitive, would be less sensitive to NSAID inhibition as well. In order to investigate this question, we compared salicylate effects on heterologously expressed WT and S1107R TRPM7 channels in HEK293 cells. We observed a robust and reversible inhibition of WT TRPM7 currents (Figures 7C,D), whereas S1107R variant was not inhibited (Figures 7D,F,G). We conclude from these experiments that NSAID inhibition of TRPM7 channels reflects the interference of acidic pH with PIP_2_-channel interactions. In agreement with this, 30mM salicylate inhibition of TRPM7 channels was relieved by application of 15mM NH_4_^+^, which alkalinizes the cytosol (*n*=3, data not shown). Interestingly, the endogenous current in HEK cells was significantly less sensitive to salicylate (Figures 6A,C) than the overexpressed TRPM7 channels in the same cell type (Figures 7C,E). This difference may be due to the dependence on TRPM7 kinase resting pH in HEK cells. We recently showed that in murine macrophages isolated from TRPM7 K1646R kinase-dead mice, the pH is more alkaline than in WT, suggesting that kinase activity may make pH_i_ more acidic (Beesetty et al., 2021). WT TRPM7 overexpressing HEK cells also respond readily to acidification in propionate and 2-APB (Zhelay et al., 2018). Although we have not tested NSAIDs directly in mammalian macrophages, they are likely to acidify the cytoplasm as is the case in *Drosophila S2* macrophages (Figure 5), leading to suppressed phagocytosis. Further investigations will be required to explain the difference between native and overexpressed TRPM7 current sensitivity to NSAIDs.

## Discussion

The present study was undertaken to characterize the effects of several common NSAIDs ibuprofen, naproxen, salicylate, and acetylsalicylate on TRPM7 channels. These drugs reversibly inhibited both native (Jurkat T cells) and recombinant TRPM7 at concentrations of 3mM and higher (Figures 1–3, 5, 7). The onset of inhibition was slow, taking several minutes, and voltage-independent, making it unlikely that these drugs interact directly with the ion permeation pathway (Figures 1–3, 5, 7). In agreement with this, TRPM7 channels with the S1107R substitution in the intracellular portion (Zhelay et al., 2018) were insensitive to salicylate (Figures 7D,F,G). TRPM7 current reduction was readily reversible upon removal of the drug (Figures 1–3, 5, 7). At concentrations of 300μM and above, these NSAIDs potently and dose-dependently acidified the cytoplasm of Jurkat T cells (Figures 4, 5). Similar to channel inhibition, the onset of acidification was also slow. Since TRPM7 channels are inhibited by acidic pH (Chokshi et al., 2012b), cytosolic acidification is likely responsible for NSAID-mediated inhibition of these channels. Previously, we demonstrated that 2-APB, a widely used TRPM7 blocker, inhibits TRPM7 channels by the same mechanism (Chokshi et al., 2012a; Zhelay et al., 2018). In Jurkat T cells, TRPM7 pH_50_ is close to 7.1 in the absence of external divalent cations (RC and JAK, unpublished observations). In view of this, pH of ~7.0 would be sufficient to inhibit TRPM7 currents substantially. It should also be noted that pH dependence of TRPM7 channel activity is not constant but can vary with PIP_2_ levels in the cell (Zhelay et al., 2018).

The observation that TRPM7 current reduction by acetylsalicylic acid (aspirin) was fully reversible at 10mM (seen in three out of four cells tested, Figure 5), suggested that COX enzyme inactivation did not underlie this effect, since unlike other NSAIDs, aspirin binds and covalently modifies COX enzymes (Fitzpatrick, 2004). Furthermore, we tested the drugs in S2 cells, a *Drosophila* macrophage-like cell line (Abrams et al., 1992), lacking both COX and TRPM7 orthologs. Cytosolic acidifications were elicited in these cells by naproxen and salicylate (Figure 5), demonstrating that neither COX enzyme inhibition nor presence of TRPM7 protein is required for the pH effect.

What are some likely consequences of acidification and TRPM7 channel inhibition? As a non-selective cation conductance, TRPM7 channel activity is expected to push the T-cell membrane potential toward 0mV (Chokshi et al., 2012b; Mason et al., 2012). A depolarization would be expected to diminish Ca^2+^ influx, curtailing the long-lasting Ca^2+^ elevations necessary for T-cell activation and clonal expansion (Feske et al., 2012). Ca^2+^ enters the activated T cell primarily through store-operated Ca^2+^-release activated Ca^2+^ (CRAC) channels (e.g., Lioudyno et al., 2008). In human erythroleukemia cells, Mg^2+^-inhibited cation (MIC) channels, likely TRPM7, participate in setting the membrane potential (Mason et al., 2012). Similarly, TRPM7 channels would be expected to depolarize T cells. The T-cell membrane potential is primarily determined by potassium-selective conductances (Feske et al., 2012; Papp et al., 2020). However, significant pre-activated TRPM7 currents can be measured in Jurkat T cells even without prior depletion of Mg^2+^ (Chokshi et al., 2012b) and would likely participate in moving the membrane potential away from K^+^ equilibrium potential. Depolarized T-cell membranes reduce Ca^2+^ entry and consequently diminish the activation of nuclear factor of activated T cells (NFAT) transcription factors, responsible for governing many gene expression events in T-cell activation and proliferation (Feske et al., 2012; Yeh and Parekh, 2017). For a related cation channel TRPM4, its genetic suppression in Jurkat T cells resulted in increased Ca^2+^ entry and IL2 production (Launay et al., 2004). Pharmacological blockade of TRPM7 channels resulted in increased IL2 receptor expression and higher number of regulatory T cells in mice (Mendu et al., 2020). Based on these examples, the inhibition of TRPM7 channels by NSAIDs would be expected to increase Ca^2+^ influx, NFAT activation, IL2 secretion and T-cell proliferation. It remains to be determined, however, what the overall NSAID effect on the membrane potential is, given that other pH-sensitive conductances, such as Kv1.3, are likely to be affected (Deutsch and Lee, 1989). In this context, it is noteworthy that pharmacological inhibition of Kv1.3 has been explored as immunomodulatory therapy for various disease states (e.g., Beeton et al., 2001; Schmalhofer et al., 2002; Rangaraju et al., 2009). In the final analysis, the action of NSAIDs on T-cell membrane potential and calcium signaling will depend on the interplay between activity of various channels and their relative pH sensitivity as well as the effects of NSAIDs on Ca^2+^ metabolism reported previously (e.g., Weiss et al., 2001; Chen et al., 2010; Villalonga et al., 2010; Munoz et al., 2011).

During repeated applications of the drugs, the inhibitory effect on TRPM7 currents increased in potency, what we refer to as use-dependence (Figures 7A,B,E). Repeated treatment of intact cells with NSAIDs, however, did not result in progressively more cytoplasmic acidification and, therefore, could not explain the use-dependence of channel blockade (Supplementary Figure S1). TRPM7 channels are inhibited by Mg^2+^ in a use-dependent fashion: applications of the same concentration of Mg^2+^ to inside-out patches resulted in progressively more potent inhibition, a form of sensitization to Mg^2+^ (Chokshi et al., 2012c). This use-dependence of Mg^2+^ inhibition was similar to what was observed with propionate (Zhelay et al., 2018), and we explain it by gradual depletion of PIP_2_ from channel vicinity and increased potency of cations in disrupting PIP_2_-channel interactions. In agreement with this, propionate-induced channel inhibition showed use-dependence only in whole-cell, which favors PIP_2_ loss but not in perforated-patch recording configuration, which preserves PIP_2_ (Zhelay et al., 2018). The extent of channel inhibition was drastically increased in cells depleted of PIP_2_ by VSP expression (Figure 6). S1107R TRPM7 channels, which are less sensitive to inhibition by protons, were insensitive to salicylate, confirming that cytosolic acidification is responsible for the observed current inhibition (Figure 7; Zhelay et al., 2018). Whether the extent of cytosolic acidification depends on the levels of PIP_2_ in the cell membrane remains to be examined.

In T cells, significant PIP_2_ depletion can occur during T cell receptor (TCR) engagement, when phospholipase C gamma is activated and hydrolyzes PIP_2_ (Kane et al., 2000). The increasing potency of block with repeated application may be relevant for long-term administration of these drugs, prescribed in gout and rheumatoid arthritis (Rider and Jordan, 2010) and for specific NSAIDs with long pharmacokinetic half-life, such as naproxen (Sugar et al., 2019). In AERD, where long-term aspirin regiments are assigned to patients as a desensitization therapy (Williams and Woessner, 2008), the use-dependence of NSAID effects may also become significant.

In the present study, we confined our analysis to NSAID inhibition of TRPM7 channels in the absence of extracellular divalent cations (Ca^2+^ and Mg^2+^). In their presence, addition of NSAIDs resulted in a reduction in tonic voltage-dependent block by divalent cations and change in the I–V relation due to the chelating action of these weak acids (data not shown; Kozak et al., 2002; Kozak and Cahalan, 2003).

We found that significant cytosolic acidification was evident at concentrations of NSAIDs lower than those required for TRPM7 channel inhibition. Thus, at 300μM and 1mM, cytosolic acidification was observed, but the channel activity was not inhibited at these concentrations. An important difference between these assays, however, was that in whole-cell patch clamp we used 1mM internal HEPES buffer which is not present in intact cells during pH measurement. The presence of HEPES, as well as weak acid glutamate, would be expected to counteract (buffer) cytosolic acidifications induced by NSAIDs. It is likely therefore, that in an intact cell, TRPM7 channels would be inhibited by lower NSAIDs concentrations, routinely achieved in blood plasma under NSAID regiments (Jung and Schwartz, 1994; Huntjens et al., 2006; Capone et al., 2007a,b). It is also likely that other ion channels sensitive to cytosolic acidification, such as TRPM3, Kir4.1, and connexins would be affected by these drugs (Pethő et al., 2020).

The focus of this study is on the acute and not long-term effects of NSAIDs on cellular pH. We also tested long-term naproxen and salicylate exposure on Jurkat T-cell viability and found that at concentrations sufficient to inhibit TRPM7, there was a significant cytotoxicity over a 24-h time period. Thus, at 3mM, the mean cell viability dropped to ~70% and at 10mM, to ~50% (Supplementary Figure S1B). By contrast, 10mM salicylate effect was modest, reaching ~85% viability (Supplementary Figure S1C). It has been reported that naproxen at 0.1–0.4mM interfered with proliferation of seal lymphocytes without causing apoptosis (Kleinert et al., 2018). Further experiments will be required to elucidate if long-term NSAID cytotoxicity and immunotoxicity are due to acidification or other mechanisms.

## Data Availability Statement

The raw data supporting the conclusions of this article will be made available by the authors, without undue reservation.

## Author Contributions

RC, OB, and TZ performed the experiments and analyzed the data. JK conceived the study, designed the experiments, and wrote and edited the manuscript. All authors contributed to the article and approved the submitted version.

## Funding

This work was funded by grants 1R15AI090613, 1R01AI114804, and 1R21AI144337 from the National Institute of Allergy and Infectious Diseases (to JK).

## Conflict of Interest

The authors declare that the research was conducted in the absence of any commercial or financial relationships that could be construed as a potential conflict of interest.

## Publisher’s Note

All claims expressed in this article are solely those of the authors and do not necessarily represent those of their affiliated organizations, or those of the publisher, the editors and the reviewers. Any product that may be evaluated in this article, or claim that may be made by its manufacturer, is not guaranteed or endorsed by the publisher.

## Footnotes

^1^ https://somapp.ucdmc.ucdavis.edu/pharmacology/bers/maxchelator/webmaxc/webmaxcS.htm

## Supplementary Material

Supplementary Figure S1 Effect of repeated application of ibuprofen on the degree of acidification. Effects of naproxen and salicylate on cell viability. **(A)** 10 mM ibuprofen-Na^+^ was applied to BCECF-loaded Jurkat T cells three times sequentially followed by washes. There was no change in the extent of acidification (F 490 nm/440 nm) during repeated application of the drug. **(B)** Jurkat T-cell viability was measured after a 24-hour incubation in the absence (control) or presence of 0.3, 3 and 10 mM naproxen-Na^+^. Each bar represents the percentage of cells (mean, SEM) which excluded trypan blue dye. **(C)** Mean Jurkat T-cell viability after a 24-hour incubation in the absence and presence of 0.3, 3 and 10 mM salicylate. Asterisks in **(B)** and **(C)** indicate significant differences compared to the control (Student’s paired t test).

## Abbreviations

TRP: Transient receptor potential
PIP_2_: Phosphatidylinositol 4,5-bisphosphate
VSP: Voltage-sensitive phosphatase
BCECF: 2',7'-Bis-(2-carboxyethyl)-5-(and-6)-carboxyfluorescein
EGTA: Ethylene glycol-bis(2-aminoethylether)-N,N,N',N'-tetraacetic acid
COX: Cyclooxygenase
HEPES: 4-(2-Hydroxyethyl)-1-piperazineethanesulfonic acid
