# Supplementary figures and images for: NSAIDs Naproxen, Ibuprofen, Salicylate, and Aspirin Inhibit TRPM7 Channels by Cytosolic Acidification
Source: Front Physiol. 2021 Oct 18;12:727549. doi: 10.3389/fphys.2021.727549 (PMC8558630; doi:10.3389/fphys.2021.727549)

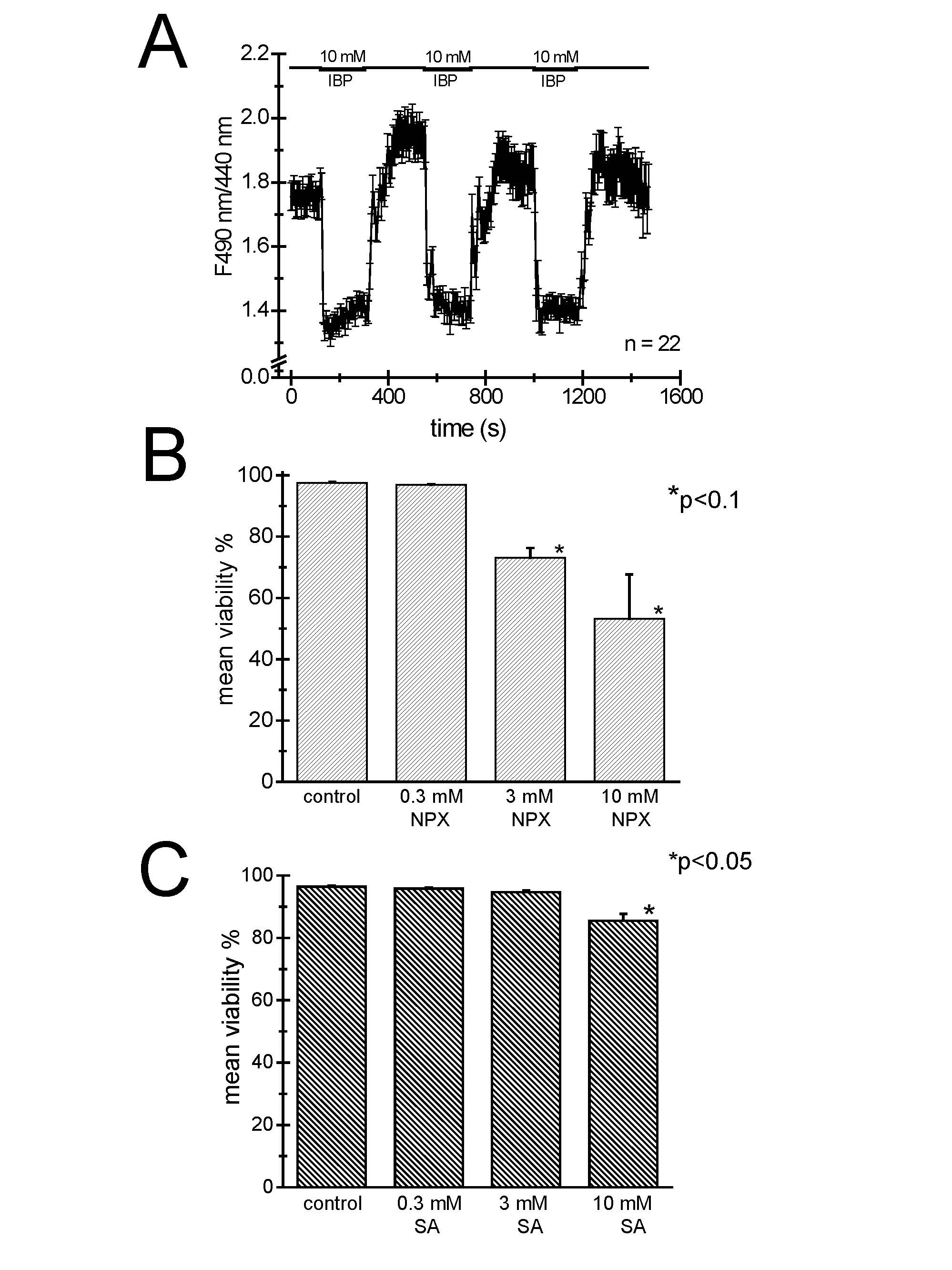

Supplement: Supplementary Figure S1 — Effect of repeated application of ibuprofen on the degree of acidification. Effects of naproxen and salicylate on cell viability. (A) 10 mM ibuprofen-Na+ was applied to BCECF-loaded Jurkat T cells three times sequentially followed by washes. There was no change in the extent of acidification (F 490 nm/440 nm) during repeated application of the drug. (B) Jurkat T-cell viability was measured after a 24-hour incubation in the absence (control) or presence of 0.3, 3 and 10 mM naproxen-Na+. Each bar represents the percentage of cells (mean, SEM) which excluded trypan blue dye. (C) Mean Jurkat T-cell viability after a 24-hour incubation in the absence and presence of 0.3, 3 and 10 mM salicylate. Asterisks in (B) and (C) indicate significant differences compared to the control (Student’s paired t test). [file Image_1.JPEG]
